# 2-Oxo-4-(thio­phen-2-yl)-1,2,5,6-tetra­hydro­benzo[*h*]quinoline-3-carbonitrile

**DOI:** 10.1107/S1600536811033915

**Published:** 2011-08-27

**Authors:** Abdullah M. Asiri, Hassan M. Faidallah, Abdulrahman O. Al-Youbi, Khalid A. Alamry, Seik Weng Ng

**Affiliations:** a Chemistry Department, Faculty of Science, King Abdulaziz University, PO Box 80203 Jeddah, Saudi Arabia; b Department of Chemistry, University of Malaya, 50603 Kuala Lumpur, Malaysia

## Abstract

In the mol­ecule of the title compound, C_18_H_12_N_2_OS, the tetra­hydro­benzo[*h*]quinoline fused-ring system is buckled owing to the ethyl­ene –CH_2_CH_2_– fragment, the benzene ring and the pyridine ring being twisted by 16.0 (1)°. The 4-substituted aromatic ring is bent away from the pyridine ring by 59.5 (2)° (for the major disordered thienyl component) in order to avoid crowding the cyanide substituent. In the crystal, two mol­ecules are linked by a pair of N—H⋯O hydrogen bonds to form a centrosymmetric dimer. The thienyl ring is disordered over two sites in a 72.7 (2):27.3 ratio.

## Related literature

For background to the anti­cancer properties of this class of compounds, see: Rostom *et al.* (2011 ▶) .
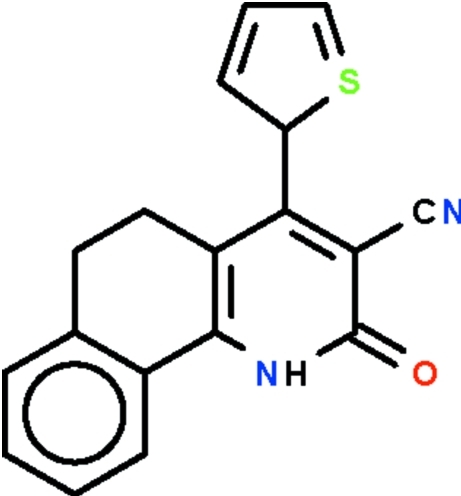


## Experimental

### 

#### Crystal data

C_18_H_12_N_2_OS
*M* *_r_* = 304.36
Triclinic, 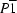

*a* = 6.9952 (3) Å
*b* = 9.1809 (4) Å
*c* = 11.1837 (5) Å
α = 93.990 (4)°
β = 95.293 (4)°
γ = 100.903 (4)°
*V* = 699.48 (5) Å^3^
*Z* = 2
Cu *K*α radiation
μ = 2.07 mm^−1^
*T* = 100 K
0.30 × 0.25 × 0.20 mm

#### Data collection

Agilent SuperNova Dual diffractometer with Atlas detector
Absorption correction: multi-scan (*CrysAlis PRO*; Agilent, 2010 ▶) *T*_min_ = 0.575, *T*_max_ = 0.682
5795 measured reflections
2740 independent reflections
2600 reflections with *I* > 2σ(*I*)
*R*_int_ = 0.020

#### Refinement

*R*[*F* ^2^ > 2σ(*F* ^2^)] = 0.035
*wR*(*F* ^2^) = 0.098
*S* = 1.07
2740 reflections
216 parameters
19 restraints
H atoms treated by a mixture of independent and constrained refinement
Δρ_max_ = 0.22 e Å^−3^
Δρ_min_ = −0.45 e Å^−3^

### 

Data collection: *CrysAlis PRO* (Agilent, 2010 ▶); cell refinement: *CrysAlis PRO*; data reduction: *CrysAlis PRO*; program(s) used to solve structure: *SHELXS97* (Sheldrick, 2008 ▶); program(s) used to refine structure: *SHELXL97* (Sheldrick, 2008 ▶); molecular graphics: *X-SEED* (Barbour, 2001 ▶); software used to prepare material for publication: *publCIF* (Westrip, 2010 ▶).

## Supplementary Material

Crystal structure: contains datablock(s) global, I. DOI: 10.1107/S1600536811033915/xu5294sup1.cif

Structure factors: contains datablock(s) I. DOI: 10.1107/S1600536811033915/xu5294Isup2.hkl

Supplementary material file. DOI: 10.1107/S1600536811033915/xu5294Isup3.cml

Additional supplementary materials:  crystallographic information; 3D view; checkCIF report

## Figures and Tables

**Table 1:** Hydrogen-bond geometry (Å, °)

*D*—H⋯*A*	*D*—H	H⋯*A*	*D*⋯*A*	*D*—H⋯*A*
N1—H1⋯O1^i^	0.89 (1)	1.97 (1)	2.851 (1)	173 (2)

Symmetry code: (i) 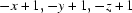.

**Fig. 1.**
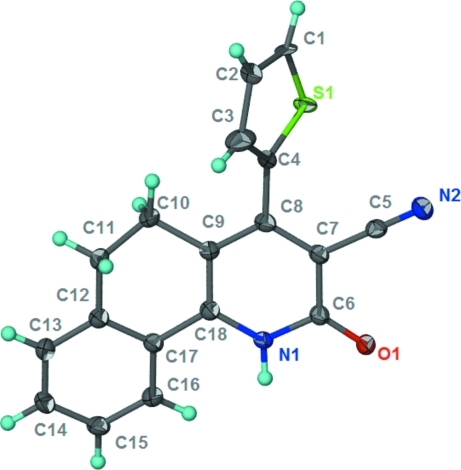
Thermal ellipsoid plot (Barbour, 2001) of C18H12N2OS at the 70% probability level; hydrogen atoms are drawn as spheres of arbitrary radius. The disorder in the thienyl ring is not shown.

## supplementary crystallographic information

### Comment

The compound (Scheme I) belongs to a series of cyano-pyridinones that have been evaluated for their anticancer properties (Rostom *et al.*, 2011). The tetrahydrobenzo[*h*]quinoline fused-ring system is buckled owing to the ethylene –CH_2_CH_2_– fragment, the benzene ring and the pyridine ring being twisted by 16.0 (1)°. The 4-subsituted aromatic ring is bent away from the pyridine ring by 59.5 (2) ° in order to avoid crowding the cyanide substituent (Fig. 1). Two molecules are linked by an N—H···O hydrogen bonds to form a centrosymmetric dimer (Table 1).

### Experimental

A mixture of thiophene-2-carbaldehyde (1.10 g, 10 mmol), 1-tetralone (1.46 g, 10 mmol), ethyl cyanoacetate (1.1 g, 10 mmol) and ammonium acetate (6.2 g, 80 mmol) in absolute ethanol (50 ml) was refluxed for 6 h. The reaction mixture was allowed to cool, and the yellow precipitate that formed was filtered, washed with water, dried and recrystallized from ethanol; m.p. 622–623 K.

### Refinement

Carbon- and nitrogen-bound H atoms were placed in calculated positions [C—H 0.95 to 0.99 *U*_iso_(H) = 1.2*U*_eq_(C)] and were included in the refinement in the riding model approximation.

The amino H atom was located in a difference Fourier map and was refined with an N—H 0.88 (1) Å restraint.

The thienyl ring is disordered over two positions in a 72.7 (2):27.3 ratio. The temperature factors of the primed atoms were set to those of the unprimed ones; the atom that is connected to the fused-ring is ordered. The anisotropic temperature factors of the disordered atoms were tightly restrained to be nearly isotropic.

### Crystal data

**Table:**

C_18_H_12_N_2_OS	*Z* = 2
*M**_r_* = 304.36	*F*(000) = 316
Triclinic, *P*1	*D*_x_ = 1.445 Mg m^−^^3^
Hall symbol: -P 1	Cu *K*α radiation, λ = 1.54184 Å
*a* = 6.9952 (3) Å	Cell parameters from 4426 reflections
*b* = 9.1809 (4) Å	θ = 4.0–74.1°
*c* = 11.1837 (5) Å	µ = 2.07 mm^−^^1^
α = 93.990 (4)°	*T* = 100 K
β = 95.293 (4)°	Prism, yellow
γ = 100.903 (4)°	0.30 × 0.25 × 0.20 mm
*V* = 699.48 (5) Å^3^	

### Data collection

**Table:**

Agilent SuperNova Dual diffractometer with Atlas detector	2740 independent reflections
Radiation source: SuperNova (Cu) X-ray Source	2600 reflections with *I* > 2σ(*I*)
mirror	*R*_int_ = 0.020
Detector resolution: 10.4041 pixels mm^-1^	θ_max_ = 74.3°, θ_min_ = 4.0°
ω scans	*h* = −7→8
Absorption correction: multi-scan (*CrysAlis PRO*; Agilent, 2010)	*k* = −11→10
*T*_min_ = 0.575, *T*_max_ = 0.682	*l* = −13→13
5795 measured reflections	

### Refinement

**Table:**

Refinement on *F*^2^	Primary atom site location: structure-invariant direct methods
Least-squares matrix: full	Secondary atom site location: difference Fourier map
*R*[*F*^2^ > 2σ(*F*^2^)] = 0.035	Hydrogen site location: inferred from neighbouring sites
*wR*(*F*^2^) = 0.098	H atoms treated by a mixture of independent and constrained refinement
*S* = 1.07	*w* = 1/[σ^2^(*F*_o_^2^) + (0.0555*P*)^2^ + 0.2491*P*] where *P* = (*F*_o_^2^ + 2*F*_c_^2^)/3
2740 reflections	(Δ/σ)_max_ = 0.001
216 parameters	Δρ_max_ = 0.22 e Å^−^^3^
19 restraints	Δρ_min_ = −0.45 e Å^−^^3^

### Fractional atomic coordinates and isotropic or equivalent isotropic displacement parameters (Å^2^)

**Table:**

	*x*	*y*	*z*	*U*_iso_*/*U*_eq_	Occ. (<1)
S1	1.02806 (12)	0.99939 (9)	0.19100 (9)	0.0171 (2)	0.727 (2)
C1	1.2328 (6)	1.0301 (6)	0.1115 (5)	0.0157 (7)	0.727 (2)
H1A	1.2578	1.1055	0.0578	0.019*	0.727 (2)
C2	1.3559 (7)	0.9300 (6)	0.1367 (5)	0.0163 (7)	0.727 (2)
H2	1.4748	0.9289	0.1022	0.020*	0.727 (2)
C3	1.2770 (14)	0.8250 (17)	0.2247 (12)	0.0299 (8)	0.727 (2)
H3	1.3373	0.7474	0.2526	0.036*	0.727 (2)
S1'	1.3139 (8)	0.8217 (10)	0.2250 (7)	0.0299 (8)	0.27
C1'	1.0531 (18)	0.9773 (13)	0.1982 (12)	0.0171 (2)	0.27
H1'	0.9394	1.0180	0.2067	0.021*	0.273 (2)
C2'	1.196 (2)	1.025 (2)	0.1248 (15)	0.0157 (7)	0.27
H2'	1.1922	1.1045	0.0750	0.019*	0.273 (2)
C3'	1.330 (2)	0.9528 (19)	0.1306 (17)	0.0163 (7)	0.27
H3'	1.4357	0.9723	0.0829	0.020*	0.273 (2)
O1	0.50080 (13)	0.56034 (11)	0.36325 (8)	0.0192 (2)	
N1	0.76813 (15)	0.61659 (12)	0.50268 (10)	0.0153 (2)	
H1	0.692 (2)	0.5628 (18)	0.5492 (14)	0.027 (4)*	
N2	0.64560 (17)	0.68980 (14)	0.08687 (10)	0.0219 (3)	
C4	1.10455 (19)	0.85666 (15)	0.25948 (11)	0.0162 (3)	
C5	0.71303 (18)	0.69445 (15)	0.18506 (12)	0.0168 (3)	
C6	0.67568 (18)	0.62164 (15)	0.38930 (12)	0.0159 (3)	
C7	0.79651 (18)	0.70041 (15)	0.30795 (12)	0.0159 (3)	
C8	0.98753 (19)	0.77471 (15)	0.34578 (12)	0.0160 (3)	
C9	1.06717 (19)	0.77150 (15)	0.46658 (12)	0.0162 (3)	
C10	1.26416 (19)	0.86240 (16)	0.52013 (12)	0.0198 (3)	
H10A	1.2453	0.9555	0.5636	0.024*	
H10B	1.3466	0.8896	0.4547	0.024*	
C11	1.36693 (19)	0.77390 (16)	0.60685 (12)	0.0182 (3)	
H11A	1.4051	0.6897	0.5610	0.022*	
H11B	1.4876	0.8387	0.6482	0.022*	
C12	1.23569 (19)	0.71478 (14)	0.69931 (12)	0.0164 (3)	
C13	1.3114 (2)	0.69892 (15)	0.81603 (12)	0.0203 (3)	
H13	1.4490	0.7224	0.8373	0.024*	
C14	1.1894 (2)	0.64951 (16)	0.90172 (12)	0.0217 (3)	
H14	1.2435	0.6397	0.9811	0.026*	
C15	0.9873 (2)	0.61428 (15)	0.87154 (12)	0.0202 (3)	
H15	0.9033	0.5831	0.9309	0.024*	
C16	0.90914 (19)	0.62486 (15)	0.75477 (12)	0.0181 (3)	
H16	0.7716	0.5983	0.7337	0.022*	
C17	1.03169 (19)	0.67444 (14)	0.66757 (11)	0.0153 (3)	
C18	0.95516 (18)	0.68759 (14)	0.54271 (11)	0.0153 (3)	

### Atomic displacement parameters (Å^2^)

**Table:**

	*U*^11^	*U*^22^	*U*^33^	*U*^12^	*U*^13^	*U*^23^
S1	0.0179 (4)	0.0139 (4)	0.0219 (3)	0.0049 (2)	0.0047 (2)	0.0097 (2)
C1	0.0132 (19)	0.0185 (8)	0.0149 (14)	−0.0033 (14)	0.0089 (11)	0.0063 (9)
C2	0.0145 (16)	0.0178 (19)	0.0197 (9)	0.0090 (9)	0.0038 (11)	0.0044 (11)
C3	0.029 (2)	0.0331 (6)	0.0259 (4)	−0.0033 (16)	0.0123 (14)	0.0043 (4)
S1'	0.029 (2)	0.0331 (6)	0.0259 (4)	−0.0033 (16)	0.0123 (14)	0.0043 (4)
C1'	0.0179 (4)	0.0139 (4)	0.0219 (3)	0.0049 (2)	0.0047 (2)	0.0097 (2)
C2'	0.0132 (19)	0.0185 (8)	0.0149 (14)	−0.0033 (14)	0.0089 (11)	0.0063 (9)
C3'	0.0145 (16)	0.0178 (19)	0.0197 (9)	0.0090 (9)	0.0038 (11)	0.0044 (11)
O1	0.0129 (4)	0.0267 (5)	0.0170 (5)	0.0003 (4)	0.0007 (3)	0.0071 (4)
N1	0.0134 (5)	0.0190 (6)	0.0137 (5)	0.0012 (4)	0.0029 (4)	0.0054 (4)
N2	0.0192 (6)	0.0287 (7)	0.0182 (6)	0.0045 (5)	0.0013 (5)	0.0070 (5)
C4	0.0159 (6)	0.0181 (7)	0.0138 (6)	0.0011 (5)	0.0005 (5)	0.0050 (5)
C5	0.0132 (6)	0.0189 (7)	0.0192 (7)	0.0030 (5)	0.0043 (5)	0.0055 (5)
C6	0.0141 (6)	0.0182 (6)	0.0160 (6)	0.0040 (5)	0.0015 (5)	0.0033 (5)
C7	0.0148 (6)	0.0191 (7)	0.0147 (6)	0.0043 (5)	0.0026 (5)	0.0052 (5)
C8	0.0159 (6)	0.0174 (6)	0.0163 (6)	0.0049 (5)	0.0038 (5)	0.0044 (5)
C9	0.0147 (6)	0.0177 (6)	0.0158 (6)	0.0015 (5)	0.0022 (5)	0.0036 (5)
C10	0.0176 (6)	0.0222 (7)	0.0169 (6)	−0.0030 (5)	0.0010 (5)	0.0041 (5)
C11	0.0139 (6)	0.0220 (7)	0.0168 (6)	−0.0006 (5)	0.0004 (5)	0.0023 (5)
C12	0.0177 (6)	0.0149 (6)	0.0157 (6)	0.0015 (5)	0.0007 (5)	0.0009 (5)
C13	0.0190 (7)	0.0206 (7)	0.0186 (7)	−0.0007 (5)	−0.0024 (5)	0.0020 (5)
C14	0.0268 (7)	0.0212 (7)	0.0148 (6)	0.0005 (6)	−0.0030 (5)	0.0035 (5)
C15	0.0242 (7)	0.0201 (7)	0.0152 (6)	−0.0001 (5)	0.0037 (5)	0.0037 (5)
C16	0.0172 (6)	0.0191 (7)	0.0170 (6)	0.0009 (5)	0.0018 (5)	0.0028 (5)
C17	0.0175 (6)	0.0144 (6)	0.0138 (6)	0.0026 (5)	0.0015 (5)	0.0015 (5)
C18	0.0148 (6)	0.0163 (6)	0.0151 (6)	0.0036 (5)	0.0020 (5)	0.0020 (5)

### Geometric parameters (Å, °)

**Table:**

S1—C4	1.7085 (14)	C6—C7	1.4378 (18)
S1—C1	1.743 (3)	C7—C8	1.3930 (18)
C1—C2	1.398 (6)	C8—C9	1.4175 (18)
C1—H1A	0.9500	C9—C18	1.3850 (18)
C2—C3	1.495 (15)	C9—C10	1.5156 (18)
C2—H2	0.9500	C10—C11	1.5230 (19)
C3—C4	1.377 (10)	C10—H10A	0.9900
C3—H3	0.9500	C10—H10B	0.9900
S1'—C4	1.631 (6)	C11—C12	1.5071 (17)
S1'—C3'	1.651 (19)	C11—H11A	0.9900
C1'—C2'	1.377 (18)	C11—H11B	0.9900
C1'—C4	1.427 (11)	C12—C13	1.3904 (18)
C1'—H1'	0.9500	C12—C17	1.4099 (18)
C2'—C3'	1.25 (2)	C13—C14	1.385 (2)
C2'—H2'	0.9500	C13—H13	0.9500
C3'—H3'	0.9500	C14—C15	1.393 (2)
O1—C6	1.2447 (16)	C14—H14	0.9500
N1—C18	1.3659 (17)	C15—C16	1.3856 (19)
N1—C6	1.3771 (16)	C15—H15	0.9500
N1—H1	0.885 (9)	C16—C17	1.4005 (18)
N2—C5	1.1491 (18)	C16—H16	0.9500
C4—C8	1.4770 (17)	C17—C18	1.4701 (17)
C5—C7	1.4361 (18)		
			
C4—S1—C1	92.12 (19)	C7—C8—C4	119.51 (12)
C2—C1—S1	111.6 (4)	C9—C8—C4	120.73 (12)
C2—C1—H1A	124.2	C18—C9—C8	118.65 (12)
S1—C1—H1A	124.2	C18—C9—C10	117.54 (12)
C1—C2—C3	111.6 (6)	C8—C9—C10	123.70 (12)
C1—C2—H2	124.2	C9—C10—C11	110.44 (11)
C3—C2—H2	124.2	C9—C10—H10A	109.6
C4—C3—C2	110.3 (10)	C11—C10—H10A	109.6
C4—C3—H3	124.9	C9—C10—H10B	109.6
C2—C3—H3	124.9	C11—C10—H10B	109.6
C4—S1'—C3'	90.2 (6)	H10A—C10—H10B	108.1
C2'—C1'—C4	108.7 (11)	C12—C11—C10	111.07 (11)
C2'—C1'—H1'	125.6	C12—C11—H11A	109.4
C4—C1'—H1'	125.6	C10—C11—H11A	109.4
C3'—C2'—C1'	112.7 (15)	C12—C11—H11B	109.4
C3'—C2'—H2'	123.6	C10—C11—H11B	109.4
C1'—C2'—H2'	123.6	H11A—C11—H11B	108.0
C2'—C3'—S1'	116.8 (14)	C13—C12—C17	118.92 (12)
C2'—C3'—H3'	121.6	C13—C12—C11	121.40 (12)
S1'—C3'—H3'	121.6	C17—C12—C11	119.69 (11)
C18—N1—C6	125.02 (11)	C14—C13—C12	121.08 (13)
C18—N1—H1	122.2 (12)	C14—C13—H13	119.5
C6—N1—H1	112.8 (12)	C12—C13—H13	119.5
C3—C4—C1'	110.3 (8)	C13—C14—C15	120.03 (13)
C3—C4—C8	124.6 (6)	C13—C14—H14	120.0
C1'—C4—C8	125.0 (5)	C15—C14—H14	120.0
C1'—C4—S1'	111.5 (6)	C16—C15—C14	119.84 (12)
C8—C4—S1'	123.5 (3)	C16—C15—H15	120.1
C3—C4—S1	114.4 (6)	C14—C15—H15	120.1
C8—C4—S1	120.97 (10)	C15—C16—C17	120.41 (12)
S1'—C4—S1	115.5 (3)	C15—C16—H16	119.8
N2—C5—C7	179.74 (15)	C17—C16—H16	119.8
O1—C6—N1	120.39 (11)	C16—C17—C12	119.65 (12)
O1—C6—C7	124.73 (12)	C16—C17—C18	122.26 (12)
N1—C6—C7	114.88 (11)	C12—C17—C18	118.08 (11)
C8—C7—C5	121.69 (11)	N1—C18—C9	119.93 (12)
C8—C7—C6	121.52 (12)	N1—C18—C17	118.58 (11)
C5—C7—C6	116.76 (11)	C9—C18—C17	121.49 (12)
C7—C8—C9	119.75 (12)		
			
C4—S1—C1—C2	0.8 (4)	S1—C4—C8—C7	59.63 (16)
S1—C1—C2—C3	0.0 (8)	C3—C4—C8—C9	61.7 (7)
C1—C2—C3—C4	−1.1 (11)	C1'—C4—C8—C9	−119.9 (7)
C4—C1'—C2'—C3'	0(2)	S1'—C4—C8—C9	58.6 (4)
C1'—C2'—C3'—S1'	−2(2)	S1—C4—C8—C9	−120.06 (13)
C4—S1'—C3'—C2'	2.4 (17)	C7—C8—C9—C18	3.23 (19)
C2—C3—C4—C1'	1.5 (11)	C4—C8—C9—C18	−177.08 (12)
C2—C3—C4—C8	−179.9 (5)	C7—C8—C9—C10	−172.91 (12)
C2—C3—C4—S1'	−113 (20)	C4—C8—C9—C10	6.8 (2)
C2—C3—C4—S1	1.7 (10)	C18—C9—C10—C11	40.78 (16)
C2'—C1'—C4—C3	−0.9 (14)	C8—C9—C10—C11	−143.04 (13)
C2'—C1'—C4—C8	−179.6 (9)	C9—C10—C11—C12	−52.44 (15)
C2'—C1'—C4—S1'	1.8 (13)	C10—C11—C12—C13	−147.28 (13)
C2'—C1'—C4—S1	−177 (9)	C10—C11—C12—C17	32.72 (17)
C3'—S1'—C4—C3	64 (19)	C17—C12—C13—C14	−2.3 (2)
C3'—S1'—C4—C1'	−2.3 (10)	C11—C12—C13—C14	177.69 (13)
C3'—S1'—C4—C8	179.1 (7)	C12—C13—C14—C15	0.2 (2)
C3'—S1'—C4—S1	−2.2 (8)	C13—C14—C15—C16	1.8 (2)
C1—S1—C4—C3	−1.5 (6)	C14—C15—C16—C17	−1.6 (2)
C1—S1—C4—C1'	2(8)	C15—C16—C17—C12	−0.5 (2)
C1—S1—C4—C8	−180.0 (2)	C15—C16—C17—C18	−179.92 (12)
C1—S1—C4—S1'	1.3 (4)	C13—C12—C17—C16	2.4 (2)
C18—N1—C6—O1	−175.77 (12)	C11—C12—C17—C16	−177.59 (12)
C18—N1—C6—C7	4.57 (19)	C13—C12—C17—C18	−178.11 (12)
O1—C6—C7—C8	175.70 (13)	C11—C12—C17—C18	1.90 (18)
N1—C6—C7—C8	−4.65 (19)	C6—N1—C18—C9	−0.6 (2)
O1—C6—C7—C5	−6.4 (2)	C6—N1—C18—C17	178.47 (11)
N1—C6—C7—C5	173.28 (11)	C8—C9—C18—N1	−3.49 (19)
C5—C7—C8—C9	−176.88 (12)	C10—C9—C18—N1	172.89 (12)
C6—C7—C8—C9	0.9 (2)	C8—C9—C18—C17	177.49 (11)
C5—C7—C8—C4	3.42 (19)	C10—C9—C18—C17	−6.14 (19)
C6—C7—C8—C4	−178.75 (12)	C16—C17—C18—N1	−16.20 (19)
C3—C4—C8—C7	−118.6 (7)	C12—C17—C18—N1	164.33 (12)
C1'—C4—C8—C7	59.8 (7)	C16—C17—C18—C9	162.84 (13)
S1'—C4—C8—C7	−121.7 (4)	C12—C17—C18—C9	−16.63 (19)

### Hydrogen-bond geometry (Å, °)

**Table:**

*D*—H···*A*	*D*—H	H···*A*	*D*···*A*	*D*—H···*A*
N1—H1···O1^i^	0.89 (1)	1.97 (1)	2.851 (1)	173 (2)

Symmetry codes: (i) −*x*+1, −*y*+1, −*z*+1.
